# Characterization of three-dimensional cancer cell migration in mixed collagen-Matrigel scaffolds using microfluidics and image analysis

**DOI:** 10.1371/journal.pone.0171417

**Published:** 2017-02-06

**Authors:** María Anguiano, Carlos Castilla, Martin Maška, Cristina Ederra, Rafael Peláez, Xabier Morales, Gorka Muñoz-Arrieta, Maite Mujika, Michal Kozubek, Arrate Muñoz-Barrutia, Ana Rouzaut, Sergio Arana, José Manuel Garcia-Aznar, Carlos Ortiz-de-Solorzano

**Affiliations:** 1 Laboratory of Preclinical Models and Analytical Tools, Division of Solid Tumors and Biomarkers, Center for Applied Medical Research and CIBERONC, Pamplona, Navarra, Spain; 2 Centre for Biomedical Image Analysis, Faculty of Informatics, Masaryk University, Brno, Czech Republic; 3 Biodevices and MEMS group, Water and Health Division, CEIT and TECNUN University of Navarra, Donostia – San Sebastián, Gipuzkoa, SPAIN; 4 Bioengineering and Aerospace Engineering Department, Universidad Carlos III de Madrid, Leganes, Madrid; 5 Biomedical Engineering Division, Instituto de Investigación Sanitaria Gregorio Marañón, Madrid, Spain; 6 Department of Biochemistry and Genetics, Faculty of Sciences, University of Navarra, Pamplona, Navarra, Spain; 7 Department of Immunology and Inmunotherapy, CIMA, Pamplona, Navarra, Spain; 8 Department of Mechanical Engineering, Multiscale in Mechanical and Biological Engineering (M2BE), Aragon Institute of Engineering Research (I3A), University of Zaragoza, Zaragoza, Spain; Hungarian Academy of Sciences, HUNGARY

## Abstract

Microfluidic devices are becoming mainstream tools to recapitulate *in vitro* the behavior of cells and tissues. In this study, we use microfluidic devices filled with hydrogels of mixed collagen-Matrigel composition to study the migration of lung cancer cells under different cancer invasion microenvironments. We present the design of the microfluidic device, characterize the hydrogels morphologically and mechanically and use quantitative image analysis to measure the migration of H1299 lung adenocarcinoma cancer cells in different experimental conditions.

Our results show the plasticity of lung cancer cell migration, which turns from mesenchymal in collagen only matrices, to lobopodial in collagen-Matrigel matrices that approximate the interface between a disrupted basement membrane and the underlying connective tissue. Our quantification of migration speed confirms a biphasic role of Matrigel. At low concentration, Matrigel facilitates migration, most probably by providing a supportive and growth factor retaining environment. At high concentration, Matrigel slows down migration, possibly due excessive attachment. Finally, we show that antibody-based integrin blockade promotes a change in migration phenotype from mesenchymal or lobopodial to amoeboid and analyze the effect of this change in migration dynamics, in regards to the structure of the matrix.

In summary, we describe and characterize a robust microfluidic platform and a set of software tools that can be used to study lung cancer cell migration under different microenvironments and experimental conditions. This platform could be used in future studies, thus benefitting from the advantages introduced by microfluidic devices: precise control of the environment, excellent optical properties, parallelization for high throughput studies and efficient use of therapeutic drugs.

## Introduction

The ability of cancer cells to migrate is one of the hallmarks of metastatic cancer [1]. Understanding how cancer cells interact with their microenvironment to migrate and invade the surrounding tissue, intravasate to blood or lymphatic vessels, and extravasate to create distant metastases is key to discovering efficient targets for anti-cancer therapy [2]. Migration has been traditionally studied using 2D migration assays, but these studies are of difficult interpretation since the mechanisms of cell migration and mechanosensing used by cells in 2D differ from those in 3D environments [3,4]. 3D migration assays have been performed *in vitro* using Boyden chambers and multi-well slides [5]. These experimental models recreate the three-dimensional confinement of the cells but provide little control of the internal morphological and biochemical environment, are not optimized for microscopic image acquisition and are not suitable for large high-throughput studies. Finally, migration assays have been performed *in vivo* in mouse models using intravital microscopy [6]. These highly physiological experiments are technically complex, provide limited staining options, are difficult to visualize and quantify, and allow limited pharmacological and mechanobiological manipulation.

Overcoming most of the limitations of the above-mentioned methods, the use of microfluidic platforms has opened the door to study cell migration in highly controlled 3D environments, while providing excellent optical properties for *time lapse* microscopy imaging and allowing parallelization for large high-throughput studies with efficient use of reagents. For instance, in the context of cancer cell migration, microfluidic devices have been used to study invasion from a primary tumor [7], overcoming of mechanical barriers [8], cell intravasation [9], adhesion to blood vessels [10], extravasation [11], and the effect of interstitial fluid stresses [12].

Some recent *in vitro* studies have provided relevant insights into the relationship between the mechanical and morphological properties of 3D collagen matrices and the dynamics of cell migration. Using migration chambers, Wolf *et al*. [13] studied HT1080 fibrosarcoma cell motility within 3D type I collagen scaffolds of different concentration and stiffness. The authors showed that cells move by proteolitic remodeling of the surrounding environment followed by integrin mediated mesenchymal migration with a speed that increases with decreasing collagen concentration (i.e., increased pore size). The authors also showed that amoeboid, metalloproteinase-independent migration, forced by inhibition of membrane type 1-matrix metalloproteinase 1 (MT1-MMP), rescues cell motility if the porosity of the scaffold is large enough to allow migration via cell and nuclear deformation. Chung *et al*. [14] used customized microfluidic platforms to show the inverse relationship between the motility of endothelial cells and the stiffness of the scaffold. Intrigued by the complexity of the relationship between migration and the porosity and rigidity of the matrix, Lang *et al*. [15] analyzed the migration of MDA-MB 231 breast carcinoma cells in collagen matrices of varying pore size and stiffness. They observed a biphasic behavior where cell invasion is enhanced by hydrogel stiffness, provided that the pore size is large enough, while is prevented by stiffness in small pore-sized hydrogels. Lautscham *et al*. [16] used linear 2D channels of varying widths and soft 3D collagen hydrogels to study the relationship between migration, hydrogel pore size, and the mechanical properties of the cell, i.e. contractility, adhesiveness, cell stiffness and nuclear volume. Finally, Steinwachs et al. [17] recently presented a quatitative finite-element based methodology to quantify cell generated cell traction forces in 3D polymers, and observed that those forces remain roughly unchanged regardless of the concentration and stiffness of the surrounding matrix.

It is a clinical observation that increasing concentration of collagen in human solid tumors is associated with higher incidence of metastasis [18]. This cannot be explained by morphological and mechanical properties only, and is possibly due to the modulating role of integrins and the plasticity of the cell motility phenotype, enhanced by matrix stiffness and a complex, rich microenvironment [19, 20]. In this context, the role of the composition is of special interest at the front of tumor invasion, located at the interface between the connective tissue and a disrupted basement membrane. Both are dense matrices that act as functional barriers that become disorganized during invasion, allowing the progression of primary tumor to secondary niches.

Matrigel^®^ is a complex protein mixture obtained from the extracellular matrix (ECM) of a transplantable rat chondrosarcoma. It contains proteins commonly found in the basement membrane of epithelial structures, such as laminin, collagen, fibronectin, or entactin. Matrigel is also known to contain growth factors required for cell homeostasis, differentiation, and tumor growth, such as basic fibroblast growth factor and epidermal growth factor. Due to these properties, Matrigel is widely used in cancer culture models to simulate the environment of a basement membrane [21]. Indeed, using 3D fibronectin reconstituted Matrigel only matrices, Zaman *et al*. [22] studied the role of matrix stiffness and attachment in the migration of DU-145 prostate cancer cells, in the absence or presence of integrin-blocking antibodies. Poincloux *et al*. [23] further investigated the 3D migration phenotype of MDA-MB-231 breast cancer cells in a matrix similar to the basement membrane using Matrigel only scaffolds. Furthermore, mixed with collagen, Matrigel is been thought to recapitulate the environment of the leading edge of cancer invasion, at the interface between the connective tissue and an increasingly disorganized basement membrane [20, 24, 25]. In this work, we use microfluidic devices to recapitulate the migration of H1299 lung cancer cells in mixed Matrigel-collagen hydrogels that approximate the tumor microenvironment at the leading edge of cancer invasion. We present the design of the microfluidic device that, along with a set of image analysis tools, provides a robust, reliable platform to study lung cancer cell migration in 3D environments, guarantees adequate optical properties, and is easily adaptable to high-throughput studies. We characterize both morphologically and mechanically the hydrogels, and quantify cancer cell migration as a factor of Matrigel content in the presence of serum and integrin-blocking antibodies. Finally, we describe the migrating phenotype of the cells under all the previously described experimental conditions.

## Materials and methods

### Cells and cell culture

We used the metastatic H1299 non-small lung cancer cell line. This cell line, listed in the American Type Cell Culture repository (ATCC), was created from a resected lymph node metastasis after radiation therapy. Cells were cultured in RPMI 1640 medium (GIBCO, Barcelona, Spain) supplemented with 10% fetal bovine serum (FetalClone III, Thermo Fisher Scientific, Madrid, Spain) and 1% of a combination of penicillin and streptomycin (100 units/ml). To generate a stable Green Fluorescence Protein (GFP) expressing H1299 line, cells were transfected with the plasmid pEGFP-C1 (Mountain View, CA, USA) and Fugene using a 1:3 ratio, following manufacturer instructions. After transfection, GFP-positive cells were selected by exposure to G148 antibiotic (Sigma Aldrich, Steinheim, Germany) and kept frozen in liquid nitrogen. For the migration experiments, passage three GFP-positive H1299 cells were thawed in a T75 cell culture flask containing 15 mL of complete cell culture medium. Once adhered to the bottom of the flask, cell culture medium was replaced with fresh medium. The next day, cells were transferred to a T25 bottle at a concentration of 400.000 cells per flask. Once 90% confluent, cells were detached using trypsin-EDTA (GIBCO, Barcelona, Spain), and resuspended in fresh serum containing medium.

### Design and fabrication of microfluidic devices

**Fig 1:**
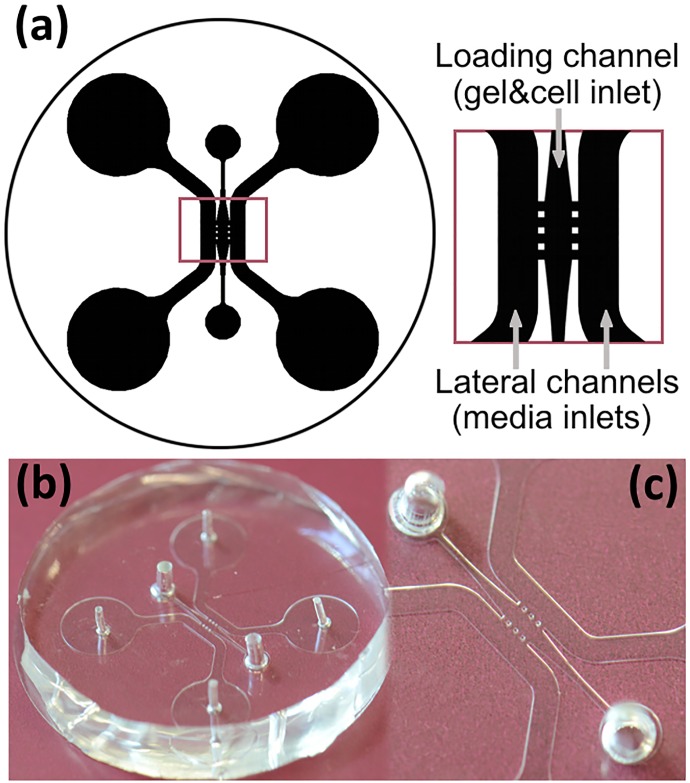
Microfluidic devices. (a) Scheme of the device. (b) General view of a polydimethylsiloxane device. (c) Detail of the chamber that contains the hydrogel and cells (center) and the channels for medium loading (side channels).

The design of the microfluidic devices was based on a previously reported concept [26]. The device consists of a central chamber 1.44mm long and 0.68mm in its widest area, connected to an inlet for hydrogel and cell insertion, and laterally connected to two 1mm wide channels for serum loading. The shape of the hydrogel chamber was optimized to ensure gentle filling, thus preventing gel breakage. The confinement of the gel and cells in the chamber was achieved by positioning of three pairs of 100μm side square posts along the merging areas of the chamber and the lateral channels. Fig 1a shows the design of the device.

Devices, shaped into 30mm diameter cylinders, were fabricated in polydimethylsiloxane (PDMS) by means of conventional replica-molding processes. The master mold was built on 4” silicon wafers by patterning using negative photoresist (SU8-100, MicroChem Co.) and standard UV-lithography techniques (see S1 File for a CAD-ready version of the mask used to fabricate the molds). The photoresist was spin coated onto the wafer at 2000 rpm providing structures up to 120μm high. Then, the wafer was soft-baked, exposed and developed following the instructions of the manufacturer. Once the master was fabricated, a two-part mixture of base and curing agents (10:1) of PDMS (Sylgard 184, Dow Corning) was poured upon it and cured at room temperature for 48 hours. The polymer provided a 10mm thick transparent replica with high quality optical properties. The silicone replica was then disassembled and cut into individual devices. The inlets and outlets were punched by 1.5mm diameter (gel insertion inlet) and 3.5mm (medium insertion inlet) needles. Finally the microfluidic structure was bonded to 35mm coverslip-slide by means of oxygen plasma, one minute treatment at 100W (Femto, Diener Electronic). The devices, shown in Fig 1b and 1c, were 40mm in diameter and 7.5mm height.

### Fabrication of hydrogels

Hydrogels were fabricated from a stock of rat tail type I collagen (BD Bioscience, San Jose, USA), diluted to the final desired collagen concentration using deionized water, 10x phosphate buffered saline (PBS), and 0.5 NaOH solution. Three types of hydrogels were prepared, one made of collagen type I and two other made of collagen type I mixed with Matrigel^®^ (BD Bioscience, San Jose, USA) (hereby Matrigel) at two different concentrations. We refer to them as hydrogels type **C** (2mg/mL collagen, no Matrigel), **CM** (2mg/mL of collagen, 2mg/mL of Matrigel), and **CM+** (2mg/mL of collagen, 4mg/mL of Matrigel), based on the increasing ratio of Matrigel to collagen. The temperature of all the components was kept at 4°C during the entire hydrogel fabrication process. When preparing **C** hydrogels, we first prepared a mixture of 10x PBS, NaOH, and water and then added collagen type I to the desired concentration. In the case of **CM** and **CM+** hydrogels, we first mixed 10x PBS, NaOH, and water, and then added type I collagen and Matrigel to the desired concentrations. The pH of all the resulting hydrogels was 7.

For control experiments in Boyden chambers, Matrigel only hydrogels (**M** and **M+**) were fabricated following the formulation used for hydrogels **CM** and **CM+**, respectively, replacing the volume of collagen type I with the same volume of acetic acid (0.02 N). Then water was added until reaching the required volume, in order to maintain the same final concentration of Matrigel as in **CM** and **CM+** hydrogels.

For the control experiments using collagen type I hydrogels with different cross-linking levels, hydrogels were fabricated following the protocol described for hydrogels of type **C**, adding the required volume of diluted transglutaminase (TG). Three different ratios of collagen-enzime were used, corresponding to increasing crosslinking levels: 100:1 (**TG6**), 50:1 (**TG13**) and 25:1 (**TG26**).

For the control experiments using collagen type I hydrogels with increasing levels of fibronectin, hydrogels were fabricated using the composition of crosslinked collagen hydrogels (100:1) at the level corresponding to hydrogels **TG6**. Then increasing amounts of fibronectin were added to obtain hydrogels containing 2mg/mL of collagen and 50μg/mL (**TG-F10**) or 100μg/mL (**TG-F20**) of fibronectin.

### Mechanical characterization of the gels: Rheometry

The mechanical properties of the hydrogels were measured using an Anton Paar Physica MCR-301 rotational rheometer (Anton Paar Germany, Seelbach, GmbH) of parallel-plate geometry. Samples of the hydrogels were prepared as described, kept at 4°C, and placed still in liquid phase between two 25mm diameter plates separated 500μm apart. Both plates were kept at 37°C. The measurements began 30 minutes after gelation on the rheometer. The storage and loss moduli of the hydrogels were measured in the linear regime, using Rheoplus/32 v3.31 software, after applying a relative strain γ = 0.01 (1%) at ω = 1.0 Hz. All measurements were repeated three times and averaged.

### Quantitative morphological characterization: Scanning electron microscopy

The ultrastructure of the hydrogels was imaged using scanning electron microscopy (SEM). To this end, samples of the hydrogel types were fixed in 4% glutaraldehyde in 0.1 M sodium cacodylate buffer, pH 7.3 at 4°C overnight and then post-fixed in 1% OsO_4_ phosphate buffer, pH 7.3 at 4°C for two hours. The samples were then dehydrated through ascending series of ethanol solutions (25% up to 100%) and critical-point dried using CO_2_. The hydrogels were sputter-coated with gold (Emitech K550) and multiple micrographs were obtained at selected regions using a Zeiss DSM 940A scanning electron microscope (20kV) (Karl Zeiss, Jena, Germany). The specimens were imaged at 3000× and 10000× magnification. To characterize the microstructural features of the 3D hydrogel networks, SEM images at 3000x magnification were analyzed using the free software ImageJ [27] and the plugin DiameterJ [28]. The average pore area, fiber diameter and percentage of porosity of the hydrogels were quantified from three independent sets of images for each hydrogel. First, the images were binarized using a statistical region-merging algorithm [29]. The percentage of porosity was then calculated from the areas of black (pores) and white (fibers) pixels within the binary images. To measure the average radius of the fibers in one image, DiameterJ calculates two different center-lines, one using an axial thinning algorithm developed by Zhang and Suen [30] and the other using Voronoi tessellation [31]. The length of each center-line is then averaged and the total area of fibers is divided by the average of the axially thinned and Voronoi center-line lengths, resulting in a unitless value that is equivalent to the mean fiber diameter. The diameter estimation is refined via intersection correction. To calculate the area of the pores, DiameterJ divides the total number of black pixels counted in pores by the total number of pores in the image.

### Quantitative morphological characterization: Confocal reflection microscopy

The structure of the hydrogels was analyzed at fiber level in confocal reflection microscopy (CRM) images of the hydrogels using an in-house developed software. Image stacks of the hydrogel were acquired on a Zeiss LSM 800 confocal microscope (Carl Zeiss, Jena, Germany) used in reflection mode. 512×512×50 voxel images were acquired using an oil-immersion Plan-Apochromatic 63× (1.4 NA) objective lens, with a final resolution of 0.099×0.099×0.42μm/voxel. The algorithm used to reconstruct and quantify individual collagen fibers involved three steps [32]. First, a steerable filter [33] was employed to reduce unwanted noise and enhance fiber-like structures. Next, the output of the filter was binarized using local Otsu’s thresholding to obtain a coarse mask of the whole collagen network. Finally, the individual fibers were extracted from the mask by tracing maximum ridges in its Euclidean distance map using the FIRE algorithm [34] that approximates each fiber using a chain of its medial axis points. Fiber length was computed by aggregating Euclidean distances between successive pairs of fiber points. Fiber persistence was calculated by finding their best least squares fitting exponential [34], and the pore area by computing the covering radius transform over a medial axis of the hydrogel liquid component [35].

### Quantitative morphological characterization: Diffusion

To characterize the transport of biomolecules within the hydrogels, 40kDa-RhodamineB dextrans (Sigma-Aldrich, Steinheim, Germany) were prepared in RPMI without phenol-red (Lonza, Belgium) at 250μg/mL and added in one of the media channels while RPMI without phenol-red was added in the other media channel. The diffusion phenomenon was imaged every five seconds during six hours in an inverted Zeiss AxioObserver microscope (Carl Zeiss, Jena, Germany). The images were analyzed using an in-house developed Fiji plugin. Briefly, twelve ROIs were manually selected in each hydrogel at three different positions within the central chamber (high, medium, and low) and their normalized intensity was measured for the duration of the experiment.

### Cell tracking assays in microfluidic devices

To perform these experiments, the cells were centrifuged and suspended in the hydrogels at a concentration of 1000 cells/μL. Before filled, the microdevices were pre-coated using Poly-D-lysine and sterilized under UV light during 20 minutes. Then, 15μL of the cell-containing hydrogel were taken using a p20 pipette equipped with a p200 tip. The tip was introduced in one of the inlets of the loading channel and the liquid mixture gently pushed until the central device chamber was completely filled. Then, the device was introduced in a Petri-dish with water and incubated at 37°C and 5% CO_2_ for 30 minutes. Once it was confirmed that the hydrogel had jellified in the central chamber, the lateral channels were filled with serum-free medium or 20% FBS medium, depending on the experiment, and the video sequence acquisition started.

For integrin-blocking experiments, cells were pre-incubated with anti-integrin antibodies at 10μg/mL two hours before filling the devices. Anti-β3 integrin antibody was purchased from Millipore (MAB2023Z; Billerica, USA) and Anti-β1 antibody was obtained from Abcam (AB7168, Cambridge, UK). In all the experiments, except for the controls, the lateral channel was filled with 20% FBS medium.

Microdevices were imaged in fluorescence and phase contrast microscopy using a Zeiss CellObserver SD spinning disc confocal microscope (Carl Zeiss, Jena, Germany) equipped with a dry Plan-Apochromatic 5× (0.12 NA) objective lens. 2D time-lapse videos of migrating cells fully embedded in the hydrogels were obtained by capturing images every 15 minutes during 12 hours. The images were 1388×1040 pixels wide, which corresponded to an area of 1.8×1.3mm^2^. The lateral motility of the cells, captured entirely by means of the long depth-of-field of the low magnification lens used, was then quantified as described in the following paragraph. Given that the cells, based on their morphology, were confirmed to be entirely embedded in the hydrogels, the 2D lateral motility was considered a good estimate of real 3D motility.

### 3D migration assays in Boyden chambers

To perform these experiments, the cells were centrifuged and suspended in the hydrogels at a concentration of 1000 cells/μL. The cell-containing hydrogels were plated on the upper side of 8μm pore-size transwell inserts (Corning, New York, USA) and incubated during one hour at 37°C. Cell migration was stimulated by placing cell media with 20% of serum in the bottom chamber and cell migration was allowed during 48 hours at 37°C. Afterwards, the cells were fixed in 4% formaldehyde for 15 minutes and the upper side of the insert was thoroughly wiped off with cotton swabs. The lower part of the insert was stained with 0.5% crystal violet. Images were captured using a Leica DMIL led inverted microscope (Leica Microsystems, Weztlar, Germany), with a HI Plan 10× (0.22 NA) objective lens and equipped with a Leica EC3 digital camera. At least four random fields were counted per experiment. Cell counts were normalized and plotted against migration in pure Matrigel with no Collagen (**M**). The Matrigel only hydrogels were also mechanically characterized as previously described for the Matrigel-collagen hydrogels.

### Cell survival after integrin-blocking

To support the evidence that the treatment with integrin-blocking antibodies does not alter cell viability, we compared the number of cells at the first and last frame of all videos corresponding to treatments that involved blocking one or both integrins, and averaged per type of hydrogel. The counting was done automatically using the segmentation algorithm described in the next section. This method of measuring viability is based on the fact that non-viable fluorescent cells lose their GFP expression and are not detected by the segmentation algorithm.

### Image analysis. Cell tracking

To extract 2D tracks of migrating cells within the microfluidic devices, using the acquired fluorescence time-lapse videos, a contrast limited adaptive histogram equalization filter was applied first to enhance image contrast [36]. Then, the cells were segmented by minimizing the Chan-Vese model using the graph cut approach [37]. This approach segments objects based on their mean intensity and does not rely on gradient information, thus allowing the segmentation of cells with imperfect or incomplete boundaries. A Matlab (Windows x64) executable version of the segmentation coded is provided in S1 Software along with a help file and the necessary DLL libraries. The segmented cells were associated between successive frames using a constrained nearest-neighbor approach, implemented in the CellTracker system [38]. Finally, we calculated the mean accumulated distance (MADs), the average speed of the cells, and the polarity of the tracks as described by Wu et al. [39]. Namely, the polarity measures the average magnitude of cell speed evaluated at different orientations, after re-alignment along the primary migration direction of each track, identified using the singular value decomposition (SVD) analysis of velocities of each cell. The polarity reveals the degree of anisotropy in cell’s velocities. If the velocity is isotropic, as in the case of a true random walk or a persistent random walk, the average magnitude of cell speed is equally likely in all directions. If the velocity is anisotropic, the average magnitude along the primary migration direction is substantially higher than along other directions.

### Immunofluorescence staining of focal adhesions in fixed cells for characterization of the migration phenotype

To evaluate cell attachments to 3D matrices, H1299 cells stably transfected with GFP were embedded in hydrogels as described previously and plated onto 8-well slides (Labteck, Nunc, Roskilde, Denmark). Cell migration was stimulated with RPMI medium (GIBCO, Barcelona, Spain) supplemented with 20% FetalClone III (Thermo Fisher Scientific, Madrid, Spain) and allowed to migrate during 12 hours. Afterwards, samples were rinsed with PBS and fixed in 4% PFA at 37°C for 30 minutes. Cells were permeabilized subsequently with 0.02% Triton X-100 in PBS and blockaded with 0.5% BSA in PBS. Cells were stained overnight at 4°C with the primary antibody Anti-FAK (1:300, Cell Signaling, Danvers, USA). Afterwards, samples were incubated for one hour at room temperature with the secondary antibody conjugated with AlexaFluor 555 (1:400, Invitrogen, Barcelona, Spain). Images were captured with an oil immersion 63× Plan-Apochromat objective (1.4 NA) on a Zeiss LSM 800 laser-scanning confocal microscope (Carl Zeiss, Jena, Germany). Images and Z sections were acquired using Zen 2.3 software (Carl Zeiss, Jena, Germany) and processed with Volocity (Perkin Elmer, Waltham MA, USA). Scale bars: 10μm.

### Statistical analysis

Each experiment was performed at least three times. All datasets were plotted as means ± SD using GraphPad Prism5 software. Box and whiskers plots indicate the 25-75th percentile by a box, whiskers show 5-95th percentiles and median value is represented as a red cross inside each box. Normally distributed data was analyzed using Kolmogorov-Smirnov’s and Shapiro-Wilk’s test. Results were compared by Student’s t-test or Anova One-Way analysis of variances followed by Bonferroni post-hoc test. Non-parametric distribution was analyzed using Mann-Whitney U-test. All statistical analyses, except that of MAD values were performed using SPSS 17.0 software (Chicago, IL, USA).

## Results

### Mechanical characterization of the hydrogels

Table 1 summarizes the viscoelastic properties of the three collagen-Matrigel mixed hydrogels. The storage modulus G’ increases with the amount of Matrigel, being the highest in type **CM+**, which is stiffer than **C** (p<0.005) and **CM** (p<0.05). A similar behavior can be seen for the loss modulus G”. In all three cases, G’ is higher than G”, indicating that all three hydrogel types behave as elastic solids. Furthermore, the ratio between G’ and G” increases with Matrigel concentration, meaning that the elastic solid behavior is more prevalent as the concentration of Matrigel increases.

**Table 1:** Mechanical characterization of the hydrogels.

Hydrogel	G’ avg (std)	G” avg (std)
C	9.03 (0.63)**	1.6 (0.17)**
CM	13.4 (5.58)*	1.76 (0.47)*
CM+	40.7 (8.20)	3.65 (0.45)

Average (avg) and standard deviation (std) of the storage (G’) and loss (G”) moduli, both in pascal units (Pa) of the hydrogel. The standard deviation corresponds to three repetitions of each experiment (n = 3).

** indicates very statistically significant difference of paired *t* test calculation (p<0.005) with respect to **CM+**.

* indicates a statistically significant difference (p<0.05) with respect to **CM+**.

### Morphological characterization of the hydrogels

**Fig 2:**
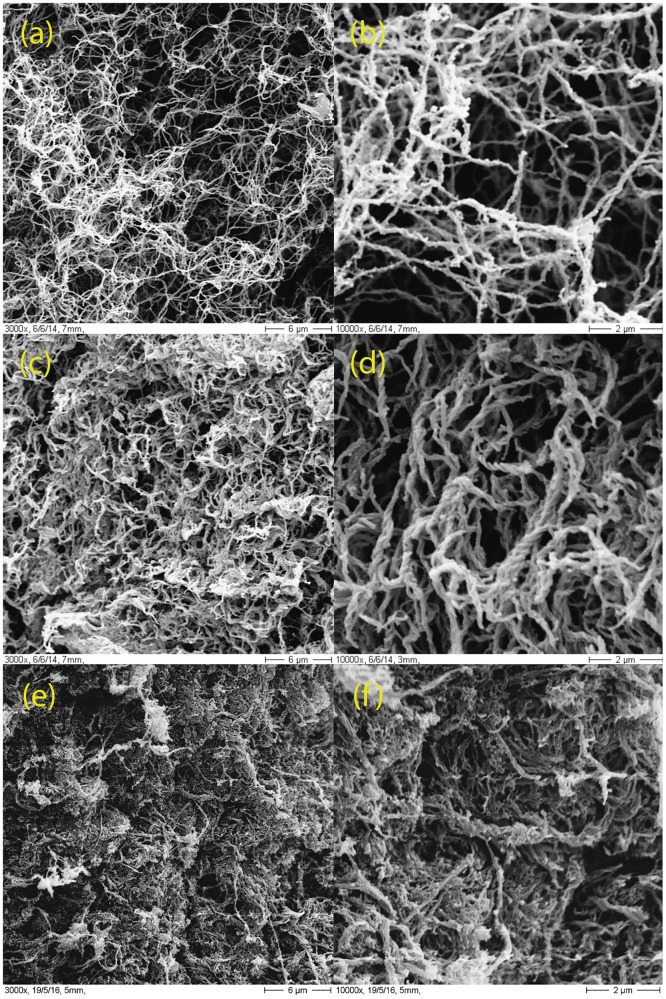
Scanning electron microscopy (SEM). Representative scanning electron micrographs of the three hydrogel types. (a, b) Type **C**; (c, d) type **CM** and (e,f) type **CM+**. The magnification shown is 3000x (left column) and 10000x (right column).

Fig 2 displays sample SEM micrographs of the three types of hydrogels. As shown, increasing the amount of Matrigel gradually increases the density of the hydrogel and the thickness of the fibers. This is in agreement with the mechanical properties described in the previous paragraph.

**Fig 3:**
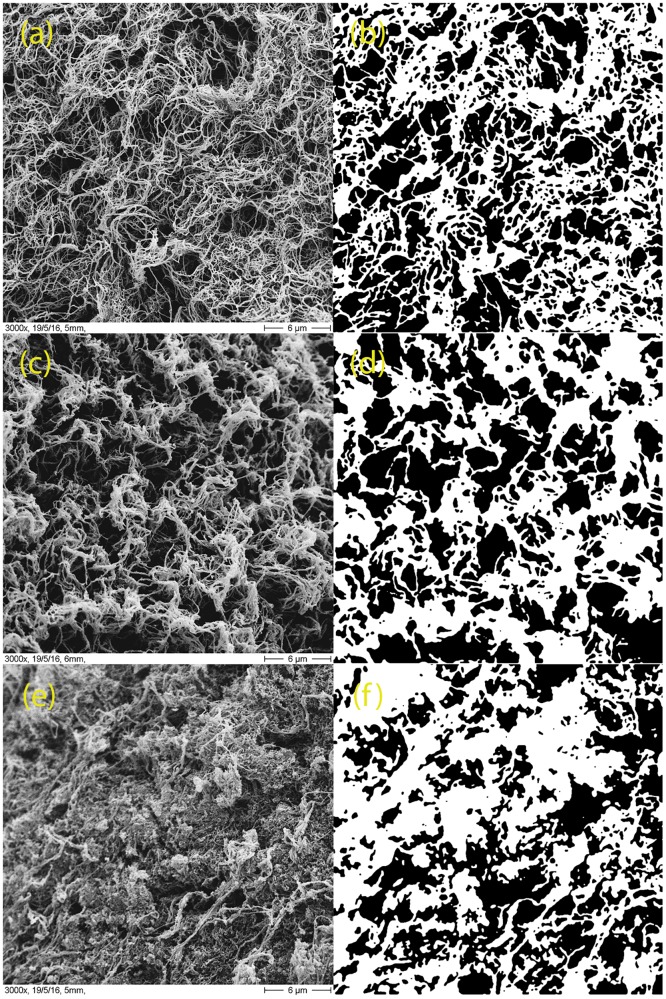
Quantification of SEM images. Representative scanning electron micrographs of one sample image of the hydrogels and their corresponding binarized images (right column). (a, b) Type **C**; (c, d) type **CM** and (e,f) type **CM+**.

**Fig 4:**
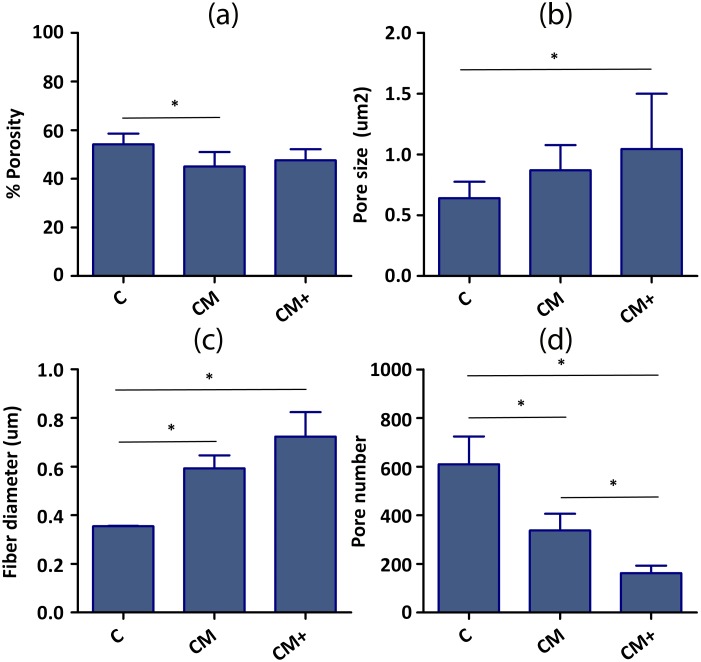
Quantification of hydrogel morphology from the SEM images. (a) % Porosity, (b) Pore size area, (c) Fiber diameter, (d) Number of pores. The number of samples used to calculate %Porosity, Fiber diameter and Number of pores is three (n = 3) since we analyzed three images from each type. The number of samples used to calculate the pore size varied between sample types, since the unit used was the pore. Namely, the n values were n = 1830 (**C**), n = 1012 (**CM**) and n = 487 (**CM+**). * Indicates statistically significant difference of non-parametric Mann-Whitney U-test (p<0.05). Pore size dataset were compared by Anova One-Way analysis of variances followed by Bonferroni post-hoc test.

The morphological parameters of the hydrogels, calculated from binarized versions of the 3000x SEM images (Fig 3) are shown in Fig 4 and S1 Table. Summarizing the results, increasing Matrigel content produces thicker fibers and causes fewer but larger pores. **CM+** hydrogels are also more heterogeneous than **CM** hydrogels, which are in turn more heterogeneous than **C** hydrogels. Due to the large number of small pores, **C** hydrogels are in average more porous than the mixed collagen-Matrigel hydrogels. It is important to emphasize that the values obtained from the SEM images represent the morphology of a dehydrated gel, and are not to be taken as real matrix values. They are shown to highlight differences between the hydrogels, or to show the level of heterogeneity of the measurements.

### Quantitative morphological characterization: Confocal reflection microscopy

**Fig 5:**
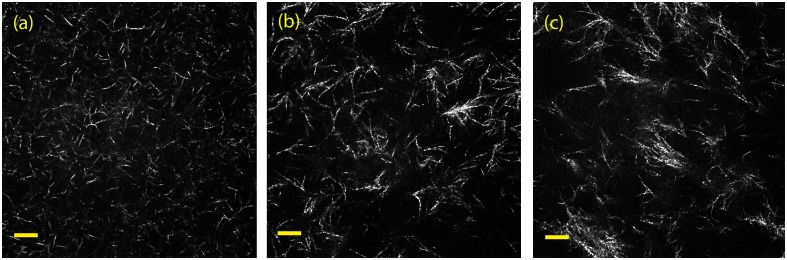
Confocal reflection microscopy. Representative confocal reflection microscopy images of the three hydrogel types. The images are single z-slices of (a) type **C**; (b) type **CM** and (c) type **CM+**. The images were taken with a 63x objective. (Scale bar: 10μm).

**Fig 6:**
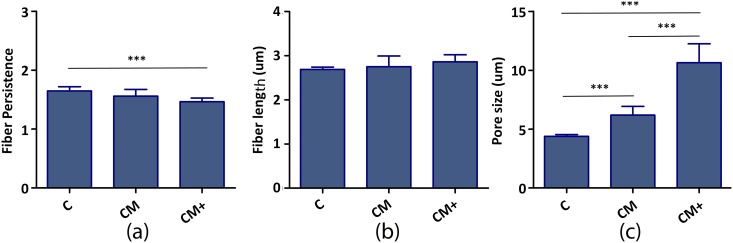
Quantification of the morphology of hydrogels C, CM and CM+ from confocal reflection microscopy images. (a) Fiber persistence, (b) Fiber length, (c) Pore size diameter. The number of samples used is nine (n = 9) since we analyzed nine sub-images from each type. *** indicates very statistically significant difference of Anova One-Way analysis of variances followed by Bonferroni post-hoc test (p<0.005).

Fig 5 shows representative confocal reflection microscopy images of hydrogels **C**, **CM** and **CM+**, and Fig 6 and S2 Table show the results of the quantification of the fibers and pore size. The results confirm the increasing pore size—and heterogeneity- caused by the addition of Matrigel, linked to a progressive shortening of fiber length.

### Quantitative morphological characterization: Diffusion

The diffusion properties of hydrogels **C**, **CM** and **CM+** were measured, as described, using a 40kDa-RhodamineB dextran (S1 Fig). The diffusion curve in hydrogel type **C**, shows how the dextran diffuses completely through the hydrogel in less than 20 minutes, while both hydrogels type **CM** and **CM+**, maintain a slow diffusive pattern at least during 360 minutes. This is consistent with the increased density and decreased porosity of hydrogels type **CM** and **CM+** compared to hydrogels of type **C**, quantified in the SEM images (see S1 Table). However, this relatively small decrease of porosity does not correspond to the large reduction in diffusivity shown in S1 Fig, pointing at other factors, such as retaining interactions between the dextran and the matrix of attachment proteins as the reason for the high retention measured in hydrogels **CM** and **CM+**.

### Quantification of cell migration

Migration experiments within the microfluidic platforms were performed using hydrogels **C**, **CM** and **CM+**, to quantify the effect of hydrogel composition, the use of serum, and the use of integrin-blocking. Three replicas were performed for each experimental condition. The total number of cells tracked for each experimental condition is summarized in Table 2.

**Table 2:** Migration experiments.

Hydrogel	C	CM	CM+
**Control**	198	180	263
**20% FBS**	383	292	354
**20% FBS + Anti-β1**	279	328	148
**20% FBS + Anti-β3**	194	316	139
**20% FBS +Anti-β1+β3**	119	280	171

Total number of cells analysed. The table indicates the type of hydrogel (**C**, **CM** and **CM+**), the presence or absence of serum (**Control** or **20% FBS**) and pre-incubation or not with integrin-blocking antibodies (**Anti-β1**, **Anti-β3** and **Anti-β1+β3**)

The control of cell survival after treatment with antibody-blocking integrins showed that cells have an almost 100% survival after treatment. The average difference between the number of cells at the beginning of the experiment and at the end of the experiment was -1 (**C**), -0.44 (**CM**) and +0.77 (**CM+**).

**Fig 7:**
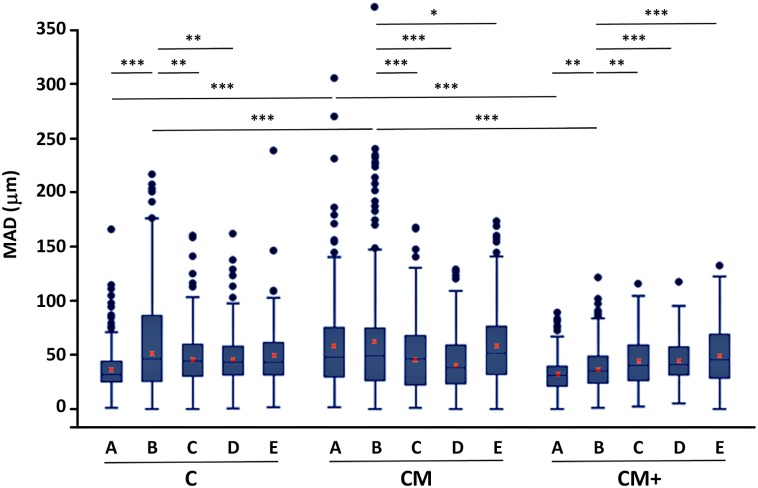
Quantification of cell migration. Box and whiskers plot of the mean accumulated distance (MAD), after 12 hours of migration hydrogel types **C**, **CM** and **CM+**, with no added chemo-attracting substance (A), using serum containing medium, (B) or after conjugation with integrin-blocking antibodies: **20% FBS + Anti-β1** (C), **20% FBS + Anti-β3** (D) and **20% FBS + Anti-β1+β3** (E). Red crosses mark the location of the mean value for each treatment and hydrogel, calculated as the pondered average of the mean value of the three replicas of each experiment. *, **,*** Indicates statistically significant difference with p<0.05, p<0.01 and p<0.001, respectively.

Fig 7 shows box-whisker plots of the results, and S3 Table contains the MAD of all the migration experiments performed. See S1 and S2 Videos for tracking results of two representative time-lapse videos of H1299 cells migrating in **CM** hydrogels (**20%FBS** and **20%FBS + Anti-β3**).

A nested factorial 3x5 ANOVA test including all hydrogels and treatments did not reveal any global significant mutual dependence among the involved variables. However, a nested ANOVA for simple effects focused on the type of hydrogel found differences between the hydrogel types (p<0.001). Posterior comparisons between the hydrogel types revealed that in the absence of serum (i.e., **Control** treatment) the cells are significantly more motile in hydrogels of type **CM** compared to hydrogels of types **C** (p<0.001) and **CM+** (p<0.001). A similar observation was found in the presence of serum (**20% FBS**), where cells in hydrogel **CM** were more motile than cells in hydrogel **C** (p<0.05) and **CM+** (p<0.001).

The impact of blocking integrins (**Anti-β1** and **Anti-β3**) was studied by means of factorial 2x2 nested ANOVAS at each hydrogel type. Global interaction was discarded when considering all three hydrogel types together. However, some pairwise interactions were found significant, as described next. In hydrogels of type **C**, a significant decrease in migration capacity—relative to **20% FBS**- was found in the presence of **Anti-β1** (p<0.01) and **Anti-β3** (p<0.01). Surprisingly, pre-incubating with both antibodies (**Anti-β1 + Anti-β3**) did not significantly reduce migration capacity. In hydrogels of type **CM**, and similar to what has been described for type **C** hydrogels, a significant reduction in migration capacity was found when using integrin-blocking antibodies, both isolated (**Anti-β1**: p<0.001; **Anti-β3**: p<0.001) and combined (**Anti-β1 + Anti-β3**: p<0.05). Finally, for type **CM+**, and in contrast with the other two hydrogels, the addition of integrin-blocking antibodies significantly increases migration capacity (**Anti-β1**: p<0.01; **Anti-β3**: p<0.001), and combining both has additive effect (**Anti-β1 + Anti-β3**: p<0.001). The analysis of the movement polarity (S2 Fig) shows that the movement is preferentially an anisotropic random walk, meaning that the cells have equal probability of migrating in any direction, but once a direction is chosen, the movement tends to advance in that direction.

To analyze the effect of Matrigel in migration, control experiments were performed using transwell Boyden chambers filled with the three mixed collagen-Matrigel hydrogels (**C**, **CM**, and **CM+**) and with two hydrogels containing only Matrigel with the same concentration as in **CM** (**M**) and **CM+** (**M+**). Please note that these control migration experiments could not be performed in microdevices because the highly viscous consistency of the Matrigel only hydrogels prevented from a proper fixation of the hydrogel within the migration chamber.

The migration results are shown in S3 and S4 Figs and S4 Table, and the mechanical properties of the Matrigel only control hydrogels are listed in S5 Table.

The migration results obtained for **C**, **CM** and **CM+** hydrogels show a similar tends to the one observed in microdevices assays. Furthermore, a significant increment in cell migration was observed in those gels that only contain Matrigel at lower concentration (**M**) compared to the hydrogels including Matrigel at high concentration (**M+**) gel. Finally, Matrigel only containing gels, **M** and **M+** clearly favor cell migration compared to their corresponding collagen containing hydrogels, **CM** and **CM+**, respectively.

The results of the migration experiments performed in collagen only hydrogels with increasing cross-linking levels and with increasing levels of fibronectin are shown in S5 Fig and S6 Table, and the morphology of those control hydrogels are shown in S6 Fig and S7 Table. Summarizing the results, we observe that hydrogels with high levels of cross-linking (**TG-13**, **TG-26**) favor cell migration compared to softer, less cross-linked hydrogels (**TG-13**) and that for a similar levels of cross-liking, increasing levels of fibronectin (**TG-F10**, **TG-F20**) slow down cell migration compared to hydrogels with no fibronectin (**TG-6**)

### Characterization of the migration phenotype

**Fig 8:**
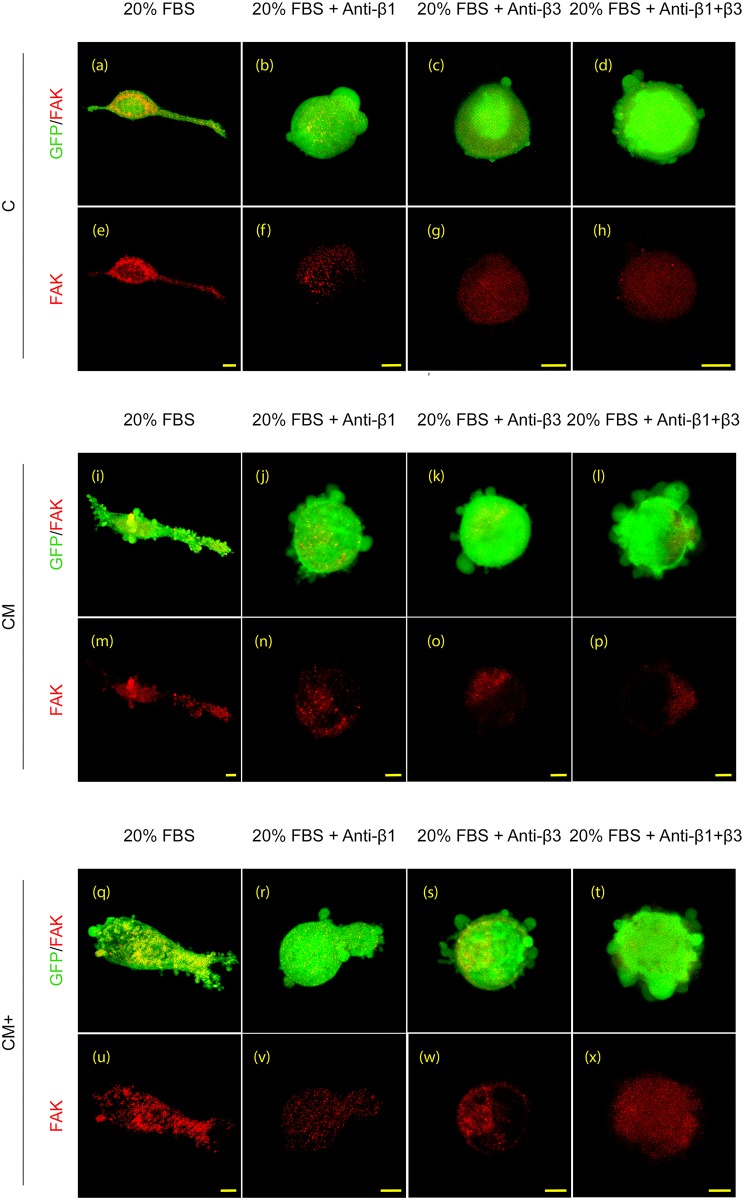
Immunophenotyping. Representative 3D renderings of confocal immunofluorescence images showing FAK (red) staining in H1299 cells embedded in the three hydrogel types (C, CM and CM+) under the treatments described in the main text. Scale bar: 10μm.

The migrating phenotype, based on the morphology of the cells was analyzed using 3D image stacks of H1299 GFP expressing cells. Examples of renderings of 3D confocal stacks of all hydrogels and treatments are shown in Fig 8.

In **C** hydrogels, the presence of serum caused a clear mesenchymal-phenotype (Fig 8a). Indeed H1299 cells showed polarization of the cell body with defined flat protrusion structures, lamellipodia at the leading edge and a rear end. Converserly, in **CM** and **CM+** hydrogels, the presence of serum caused a lobopodial phenotype (Fig 8i and 8q) since H1299 cells showed a blunt-ended cylindrical protrusion characterized by the presence of numerous short-lived membrane blebs along their surface.

Blocking β1, β3 or both surface integrins, triggered a switch to amoeboid migration phenotype, independent of the hydrogel composition (Fig 8b–8d, 8j–8l and 8r–8t). Accordingly, polarity disappeared and the cells acquired a rounded morphology accompanied by multiple small bleb-like protrusions on their surface. It should be noted that the combined blockade of β1 and β3 integrins increased the number and size of these blebs on the surface of H1299 cells compared with the single blocking treatments. This increment in the number of protruding structures seems to parallel the increase in migration ratios showed in Fig 7 and S3 Table. Regarding the cell attachment to hydrogels, the staining of focal adhesions using Focal Adhesion Kinase (FAK) antibody revealed the presence of multiple focal adhesion clusters at cell protrusions both in mesenchymal and lobopodia phenotypes. However, rounded amoeboid cells showed a faint staining of FAK antibody at cell protrusions. In contrast, it was distributed mainly homogeneously in the cytoplasm indicating a lack of cell adhesion to the matrix components.

## Discussion

H1299 lung cancer cells slowly move through collagen only (**C**) 3D hydrogels at a speed that ranges from 2.91μm/hr (control) to 4.37μm/hr if stimulated by serum. These values are similar to those reported by others using cancer cell lines (e.g., MDA-MB-231/MT1, DU-145) [22, 23]. Our analysis of migration directionality shows that H1299 cells move following an anisotropic random walk, as had been previously shown for cell migration in 3D scaffolds [39].

In the presence of Matrigel (**CM**), H1299 cells increase their migration speed, possibly due to the increased matrix stiffness and pore size of **CM** hydrogels compared to **C** hydrogels. This fact is supported by studies that show that increasing the rigidity of collagen matrices of large enough pore size increases cell migration speed [15] and is also consistent with our control experiments using collagen only hydrogels with different levels of crosslinking. Indeed, our control experiments show that cells in highly crosslinked hydrogels move faster than in softer hydrogels, due to the combined effect of the increased stiffness and increased pore size. This change in migration speed is also coincident with a change in migration phenotype. We have shown that H1299 lung cancer cells display a mesenchymal type of migration in pure collagen matrices (**C**) that turns to lobopodial in mixed collagen-Matrigel matrices (**CM** and **CM+**). This plasticity could be explained by both the composition and the mechanical properties of the hydrogels. Indeed it has been described that fibroblasts show efficient lobopodial-based motility in cell-derived matrices with linear-elastic component, similar to our mixed matrices, while show a lamellipodial phenotype (i.e., mesenchymal) in 3D collagen-only matrices [20, 25]. This switch from mesenchymal to lobopodial migration has been linked both to an increment in mesh rigidity as well as to the presence of soluble factors, heavily present in Matrigel, that are known to activate the small GTPase RhoA pathway [20]. Finally, our results also confirm that the lobopodial migrating cells in **CM** mixed matrices move faster than the mesenchymal migrating cells in collagen only matrices [20].

Surprisingly, H1299 cells in stiffer **CM+** hydrogels slow down their migration speed compared to **CM** hydrogels. This seems to contradict our previous observations, since the increased stiffness and larger pore size of **CM+** hydrogels should in principle enhance cell migration. This behavior is however consistent with our control transwell experiments in Matrigel only hydrogels that show a reduction in migration speed with increasing Matrigel concentration, regardless of the increased hydrogel stiffness. This could be explained by the increased attachment caused by Matrigel components. In fact, a similar behavior has been reported by Zaman *et al*. in matrices of reconstituted Matrigel with increasing levels of fibronectin [22] and is also visible in our own control experiments using collagen hydrogels with different amounts of fibronectin—one of the main components of Matrigel-. In these control experiments we see a decrease in migration speed in fibronectin containing collagen scaffolds compared to bare collagen hydrogels. Accordingly, there seems to be a balance between the contradictory effects of stiffness and attachment. In **CM** hydrogels, the benefits of the increased rigidity as well as an increment in pore size are more prevalent than the problems caused by the increased attachment. Contrarily, in **CM+** hydrogels the impact of increased attachment overcomes the migration benefits of increased rigidity and larger pore size. In summary, our results confirm that elements present in Matrigel promote a change in migration phenotype from mesenchymal to lobodopial. These elements, at low concentration, facilitate migration, most probably by providing a supportive and growth factor-retaining environment. However, an excess of Matrigel impedes migration due to excessive attachment and a higher confinement that impairs cell motility.

The experiments using integrin-blocking antibodies emphasize the modulating effect of the properties of the microenvironment. These experiments show that blocking one or both integrins causes a transition to amoeboid, integrin independent migration phenotype in all three hydrogel types. These results are strongly supported by other authors who observed how the blockade of MMPs or integrins triggers an amoeboid phenotype that yields efficient migration ratios in 3D lattices. Indeed, it had been shown that in situations of high confinement, and in the absence of focal adhesions, mesenchymal cells spontaneously switch to an amoeboid migration phenotype [22, 40–42]. However, The effect of this switch in the migration speed is different in the three hydrogel types, pointing again to an equilibrium between the role of integrins as attachment and traction mediators, as well as to the role of the pore size. Indeed, in collagen only (**C**) and **CM** hydrogels, blocking integrins reduces migration speed due to an ineffective attachment to the substrate that affects the traction forces required for integrin-mediated migration. Consequently, in these smaller pore sized hydrogels, the amoeboid migration phenotype is less effective than the original mesenchymal migration. Contrarily, in larger pore sized **CM+** hydrogels, the switch to amoeboid phenotype enhances migration speed, probably due to the fact that these hydrogels cause excessive attachment that is released when integrins are blocked. This supports previous observations that indicate that tumor cells moving along collagen fibers progress or are retained through adhesion depending on the density of the surrounding matrix [43] and their capability to squeeze within the mesh pores. Finally, our results nicely support the results of Zaman et al. [22] in fibronectin reconstituted Matrigel hydrogels. The authors found the expected reduction in migration speed after integrin blockade when using Matrigel with no fibronectin (representative of our low attachment **C** hydrogels) but observed a speed enhancement effect in Matrigel with an abundance of fibronectin (representative of our high attachment **CM+**) gels. Surprisingly, but consistent with our previous discussion, blocking both integrins only has an additive effect compared to blocking only one of them in **CM+** hydrogels. This points to an incomplete switch from integrin-dependent to integrin-independent movement when only one of the integrin is blocked [41, 44] and in turn seems to indicate a compensatory mechanism by which blocking one of the integrins increases the activity of the other integrin, as has been reported by Wennenberg *et al*. [45].

It is also interesting to point out that the lobopodial migration phenotypes seen in mixed hydrogels is different both morphologically and in terms of integrin dependence not only from the amoeboid type of migration [20], but also from the migration phenotype described by Poincloux et al. [23] in Matrigel only scaffolds. As described by the authors, migrating breast cancer cells show a rounded shape with an actomyosin-based uropod that generates contractile forces at the cell rear. These contractile forces are transmitted to and exert traction forces on the ECM through β1 integrins, thus pulling on the matrix in the rearward direction, generating forward movement of the cell that pushes the matrix at the front. Therefore, the lobopodial type of migration seems to be an intermediate step between a pure mesenchymal type of migration in collagen only matrices and a rounded uropod-based type of migration in Matrigel only hydrogels, due possibly to the presence of Matrigel while retaining a prevalent collagen structure.

In summary, we have characterized the migration phenotype and dynamics of H1299 NSCLC cells in matrices that mimic different tumor microenvironments, from pure collagen matrices similar to connective tissue to mixed collagen-Matrigel matrices that approximate a disorganized basement membrane at the front of cancer invasion. We have shown how the composition and mechanical properties of the environment affect the migration of the cells, free or under the effect of integrin-blocking antibodies. We have explained our results in light of what it is known about cell migration in 3D environments. Furthermore, we have described the microfluidic platform and image analysis tools that could be used, combined with the described hydrogels, to carry out new cancer and anti-cancer related studies in a robust microfluidic platform with excellent optical properties and which is easily extensible to high-throughput mode. The use of these devices would allow the study of tumoral invasion strategies in different environments as well as define efficient therapeutic anti cancer drugs.

## Supporting information

S1 Fig Diffusion study. Fluorescence intensity captured from a 40kDa-RhodamineB dextran in three parts of the hydrogel H (part of the gel close to the insertion channel), M (middle of the hydrogel), L (part of the gel in the channel opposite to the insertion channel), during 6 hours of experiment, for hydrogels type **C** (a), **CM** (b) and **CM+** (c).
(TIF)

S2 Fig Polarity plots. Analysis of the average magnitude of the speed of the cells evaluated at different orientations, after re-alignment along the primary migration direction of each track. **C** (a), **CM** (b) and **CM+** (c).
(TIF)

S3 Fig 3D Transwell experiments. Representative images used to quantify cell migration in hydrogels **C** (a), **CM** (c) and **CM+** (e), and in hydrogels made of Matrigel only with the same concentration as in **CM, M** (b) and **CM+, M+** (d). Snapshots show cell migration in the different hydrogels towards 20% FBS.
(TIF)

S4 Fig Quantification of H1299 cell migration capability in 3D Transwell experiments. Migration fold, relative to M hydrogels of all the described hydrogels, in the presence or absence of serum. The number of replicas of each experiment is 8 for 20% FBS experiments and 4 for those without serum. *** indicates very statistically significant difference of Anova One-Way analysis of variances followed by Bonferroni post-hoc test (p<0.005).
(TIF)

S5 Fig Quantification of H1299 cell migration in hydrogels with increasing levels of cross-linking or fibronectin content. A: Serum free. B: 20%FBS. The number of cells analyzed was: TG-6: 176 (A), 178 (B); TG-13: 271 (A), 303 (B); TG-26 250 (A), 231 (B); TG-F10 162 (A), 211 (B); TG-F20 164 (A), 135 (B). *** indicates very statistically significant difference of Anova One-Way analysis of variances followed by Bonferroni post-hoc test (p<0.005).
(TIF)

S6 Fig Morphological characterization of the hydrogels used in the control experiments from confocal images. Average and standard deviation (std) of the morphological measurement obtained from Confocal Reflection Microscopy images. The number of samples used to calculate the Fiber length, Fiber persistence, and Pore size is nine (n = 3) since we analyzed three sub-images from each type. * Indicates statistically significant difference of non-parametric Mann-Whitney U-test (p<0.05).
(TIF)

S1 Table Morphological characterization of the hydrogels from SEM images. Average and standard deviation (std) of the morphological measurement obtained from the SEM images. The number of samples used to calculate the %Porosity, Fiber diameter and Number of pores and is three (n = 3) since we analyzed three images from each type. The number of samples used to calculate Pore size varied between sample types, since the unit used was the pore. Namely, the n values were n = 1830 (**C**), n = 1012 (**CM**) and n = 487 (**CM+**).
(DOCX)

S2 Table Morphological characterization of the hydrogels from confocal images. Average and standard deviation (std) of the morphological measurement obtained from the Confocal Reflection Microscopy images. The number of samples used to calculate the Fiber length, Fiber persistence, and Pore size is nine (n = 9) since we analyzed nine sub-images from each type.
(DOCX)

S3 Table Migration experiments. Mean and standard error (parenthesis) of accumulated distance (in microns) after 12 hours of migration, and speed of migration (in microns per hour) in hydrogels **C**, **CM** and **CM+**, with no chemo-attracting substance (**Control**) using serum containing medium, (**20% FBS**) or after conjugation with integrin-blocking antibodies (**20% FBS + Anti-β1**, **20% FBS + Anti-β3** and **20% FBS + Anti-β1+β3**).
(DOCX)

S4 Table Transwell invasion experiments. Mean and standard error (parenthesis) of number of invading cells in **C**, **CM**, and **CM+**, hydrogels, and in hydrogels with Matrigel only at equal concentration as in **CM** (**M**, 2mg/ml) and **CM+** (**M+**, 4mg/ml). The number of replicas of each experiment is 8 for **20%FBS**, and 4 for serum free, **Control** experiments.
(DOCX)

S5 Table Mechanical characterization of the hydrogels. Average (avg) and standard deviation (std) of the storage (G’) and loss (G”) moduli, both in pascal units (Pa) of the control, Matrigel only containing hydrogels. The standard deviation corresponds to three repetitions of each experiment (n = 3).
(DOCX)

S6 Table Control experiments. Mean and standard error (parenthesis) of accumulated distance (in microns) after 12 hours of migration in collagen only hydrogels with increasing crosslinking levels **TG6**, **TG13**, and **TG26**, and with increasing levels of fibronectin **TG-F5**, **TG-F10** and **TG-F20**, with no chemo-attracting substance (**Control**) and using serum containing medium, (**20% FBS**). The numbers are average values obtain in two migration experiments.
(DOCX)

S7 Table Morphological characterization of the hydrogels used in the control experiments from confocal images. Average and standard deviation (std) of the morphological measurement obtained from Confocal Reflection Microscopy images. The number of samples used to calculate the Fiber length, Fiber persistence, and Pore size is nine (n = 3) since we analyzed three sub-images from each type.
(DOCX)

S1 Video Time-lapse videos corresponding to an experiment performed in a CM hydrogels with 20%FBS with the calculated tracks overlapped. (AVI)

S2 Video Time-lapse videos corresponding to an experiment performed in a CM hydrogels with 20%FBS + Anti-β3 with the calculated tracks overlapped. (AVI)

S1 File Microdevice design. CAD-ready version of the mask used to fabricate the molds.
(DWG)

S1 Software Segmentation software. A Matlab (Windows x64) executable version of the segmentation coded is provided in S1 Software along with a help file and the necessary DLL libraries.
(ZIP)
