# Supplementary figures and images for: Characterization of three-dimensional cancer cell migration in mixed collagen-Matrigel scaffolds using microfluidics and image analysis
Source: PLoS One. 2017 Feb 6;12(2):e0171417. doi: 10.1371/journal.pone.0171417 (PMC5293277; doi:10.1371/journal.pone.0171417)

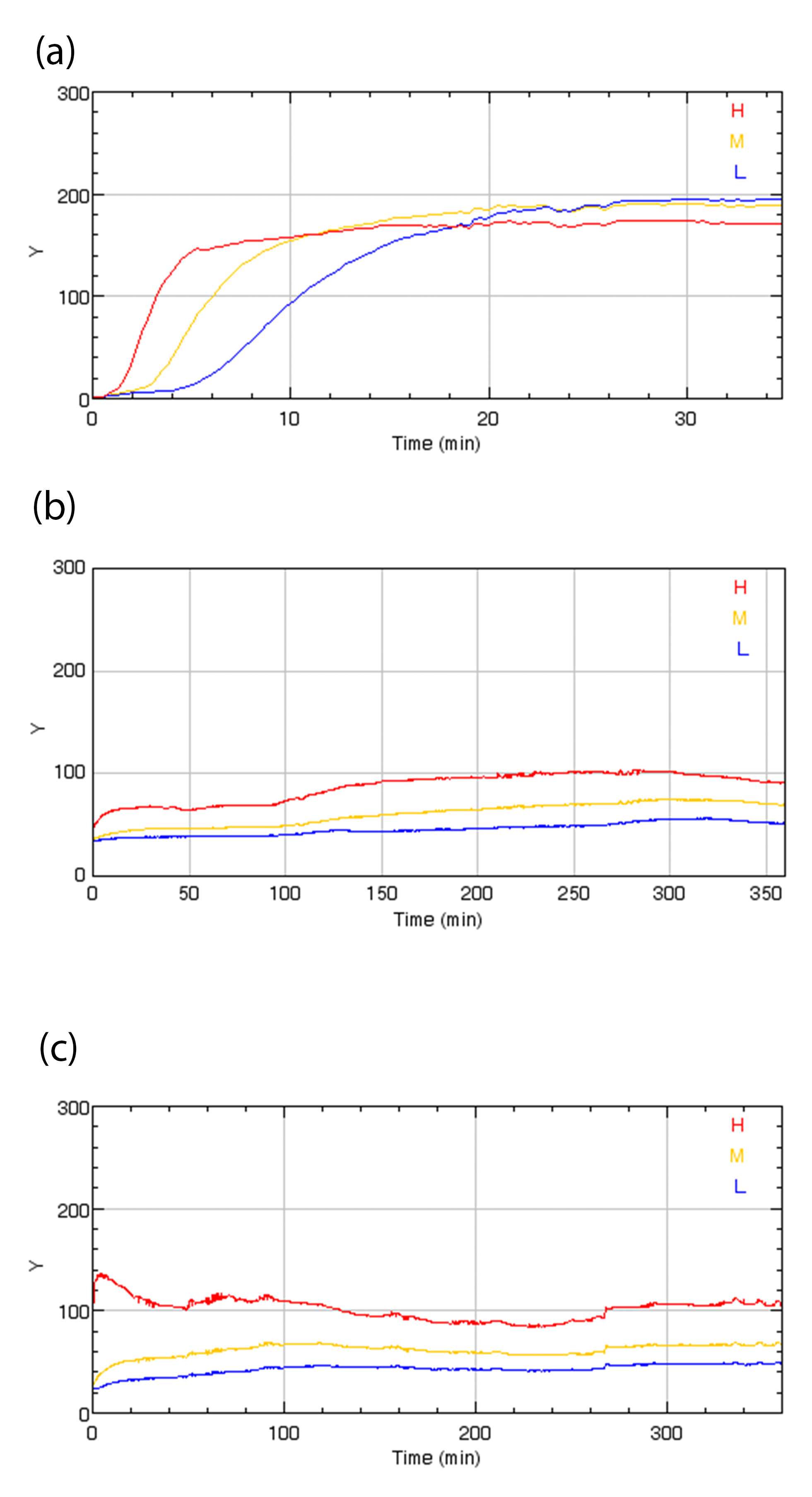

Supplement: S1 Fig — Fluorescence intensity captured from a 40kDa-RhodamineB dextran in three parts of the hydrogel H (part of the gel close to the insertion channel), M (middle of the hydrogel), L (part of the gel in the channel opposite to the insertion channel), during 6 hours of experiment, for hydrogels type C (a), CM (b) and CM+ (c). (TIF) [file pone.0171417.s001.tif]

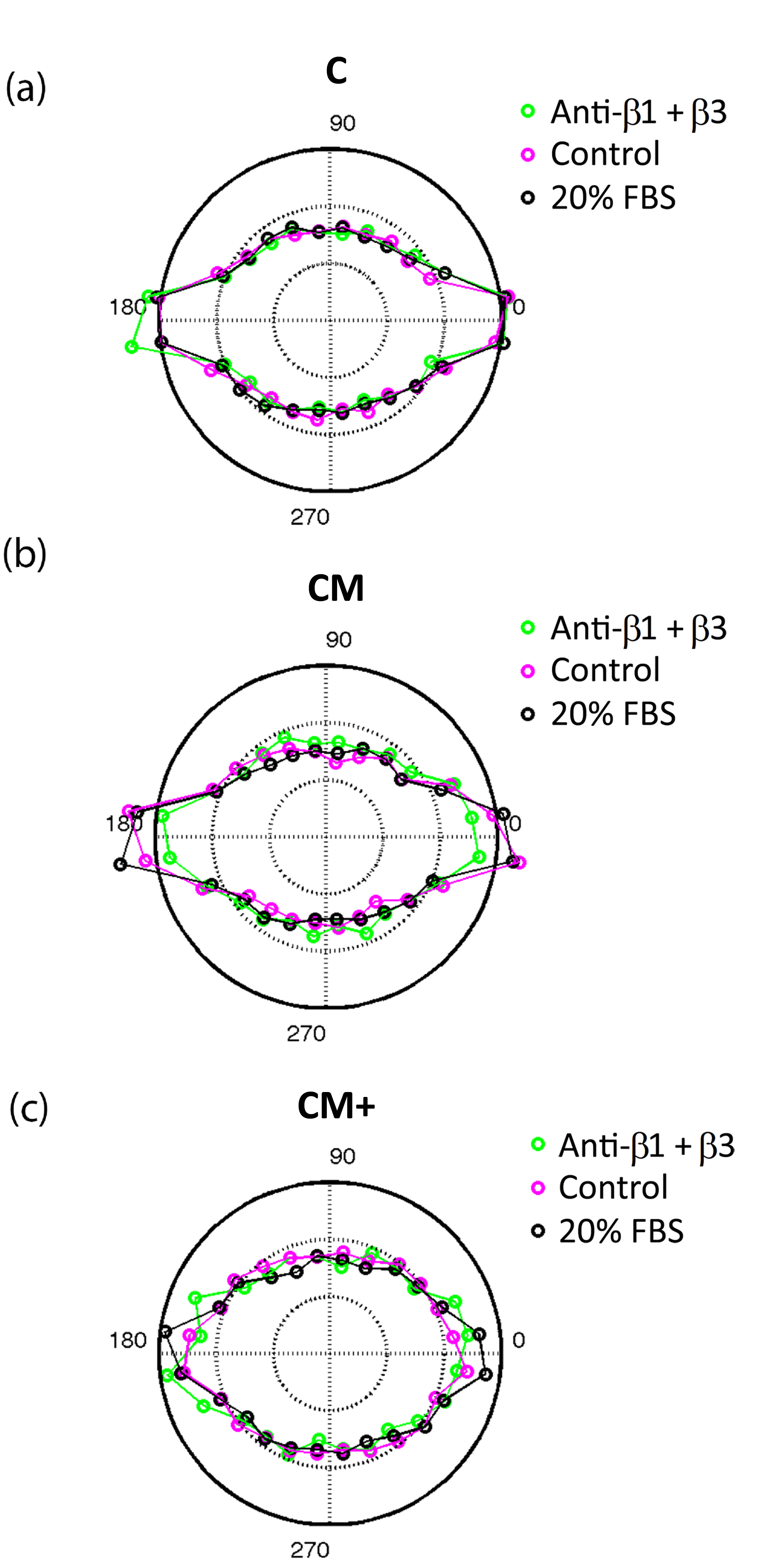

Supplement: S2 Fig — Analysis of the average magnitude of the speed of the cells evaluated at different orientations, after re-alignment along the primary migration direction of each track. C (a), CM (b) and CM+ (c). (TIF) [file pone.0171417.s002.tif]

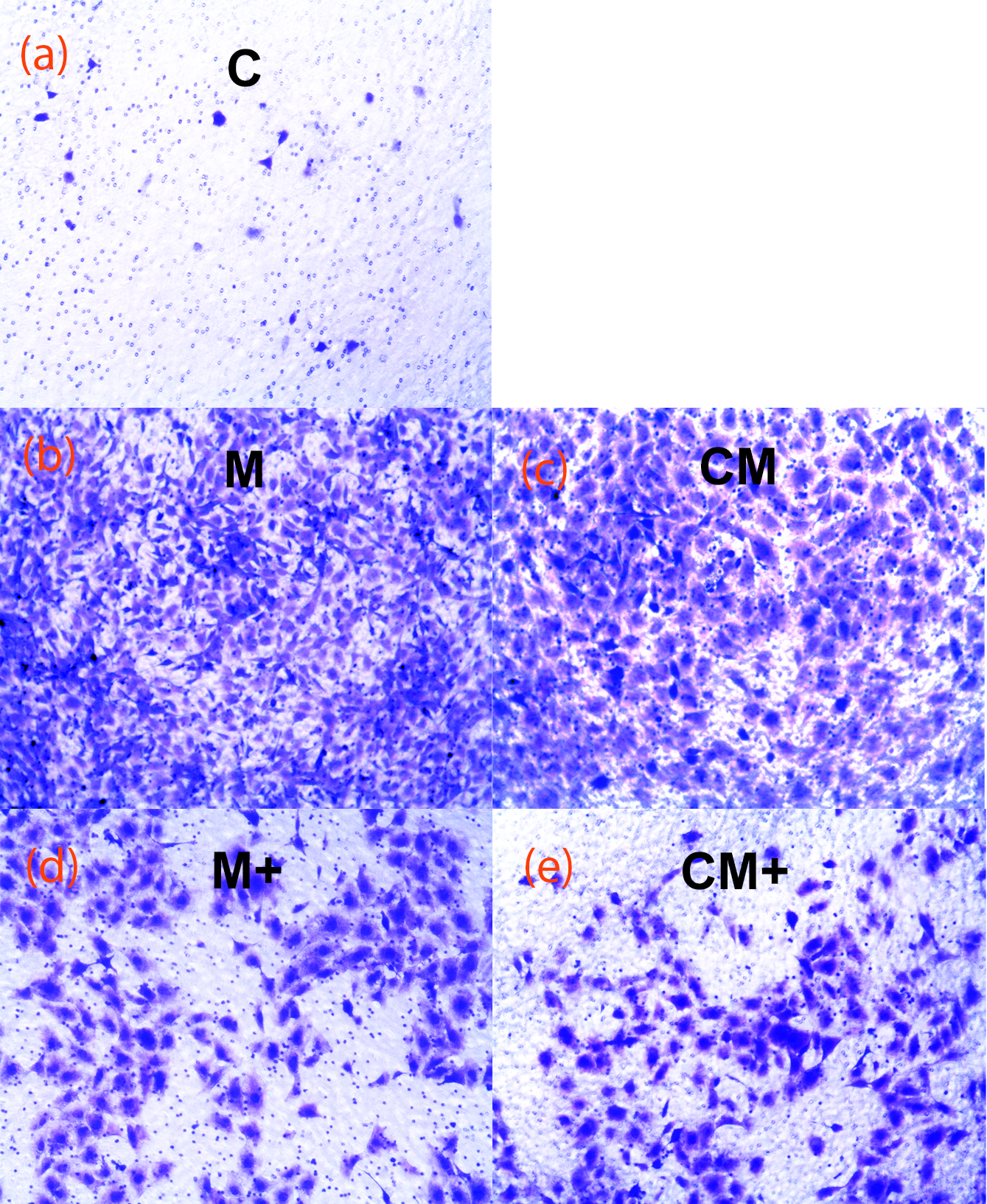

Supplement: S3 Fig — Representative images used to quantify cell migration in hydrogels C (a), CM (c) and CM+ (e), and in hydrogels made of Matrigel only with the same concentration as in CM, M (b) and CM+, M+ (d). Snapshots show cell migration in the different hydrogels towards 20% FBS. (TIF) [file pone.0171417.s003.tif]

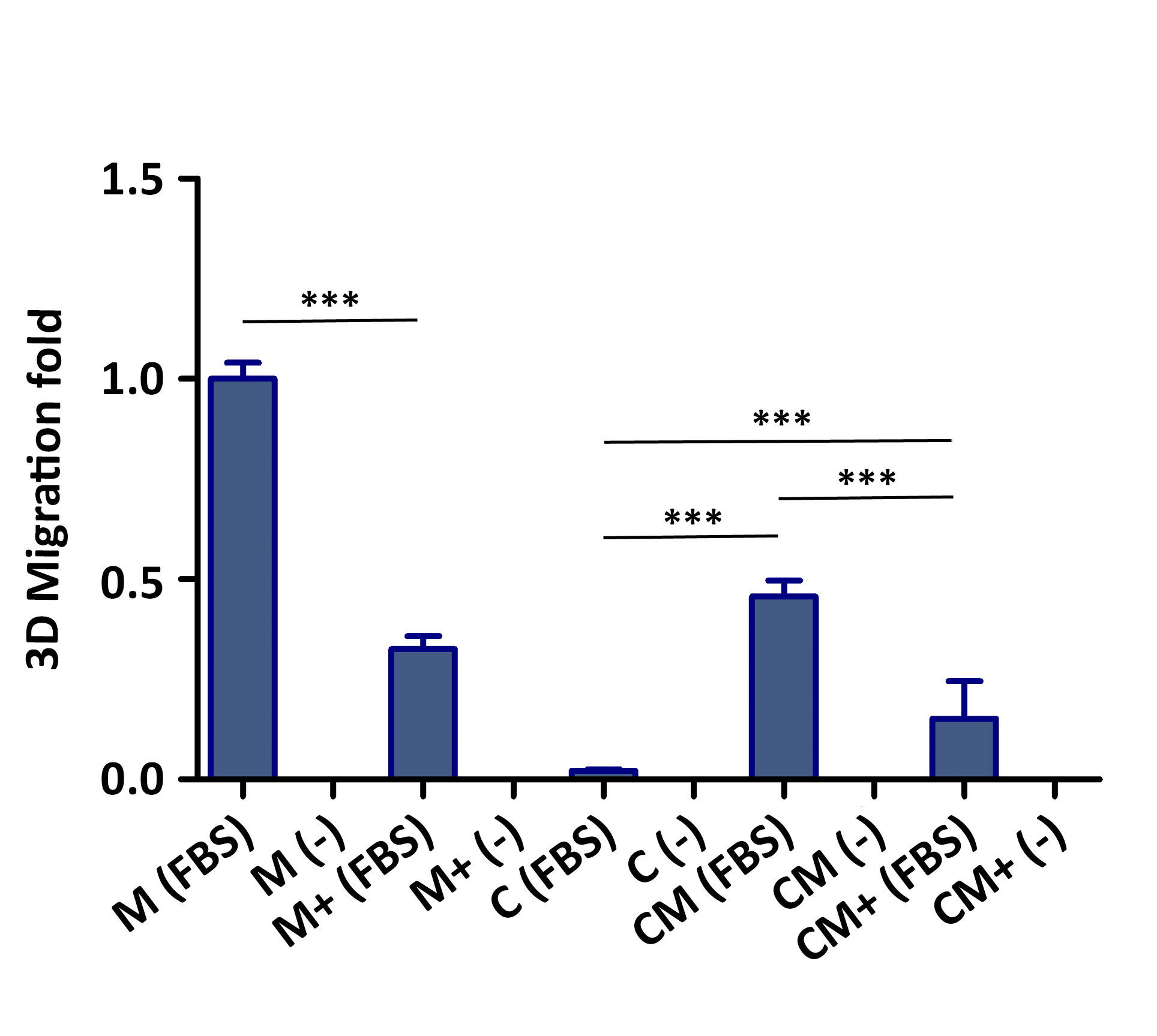

Supplement: S4 Fig — Migration fold, relative to M hydrogels of all the described hydrogels, in the presence or absence of serum. The number of replicas of each experiment is 8 for 20% FBS experiments and 4 for those without serum. *** indicates very statistically significant difference of Anova One-Way analysis of variances followed by Bonferroni post-hoc test (p<0.005). (TIF) [file pone.0171417.s004.tif]

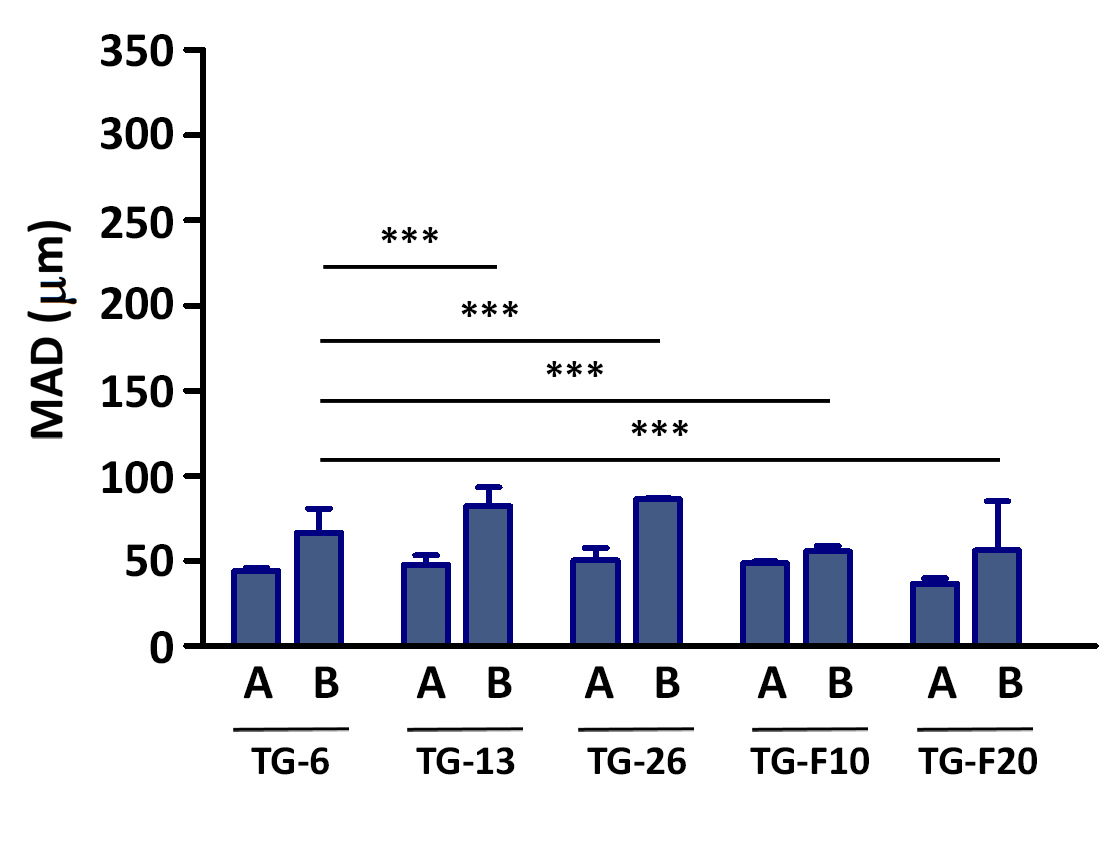

Supplement: S5 Fig — A: Serum free. B: 20%FBS. The number of cells analyzed was: TG-6: 176 (A), 178 (B); TG-13: 271 (A), 303 (B); TG-26 250 (A), 231 (B); TG-F10 162 (A), 211 (B); TG-F20 164 (A), 135 (B). *** indicates very statistically significant difference of Anova One-Way analysis of variances followed by Bonferroni post-hoc test (p<0.005). (TIF) [file pone.0171417.s005.tif]

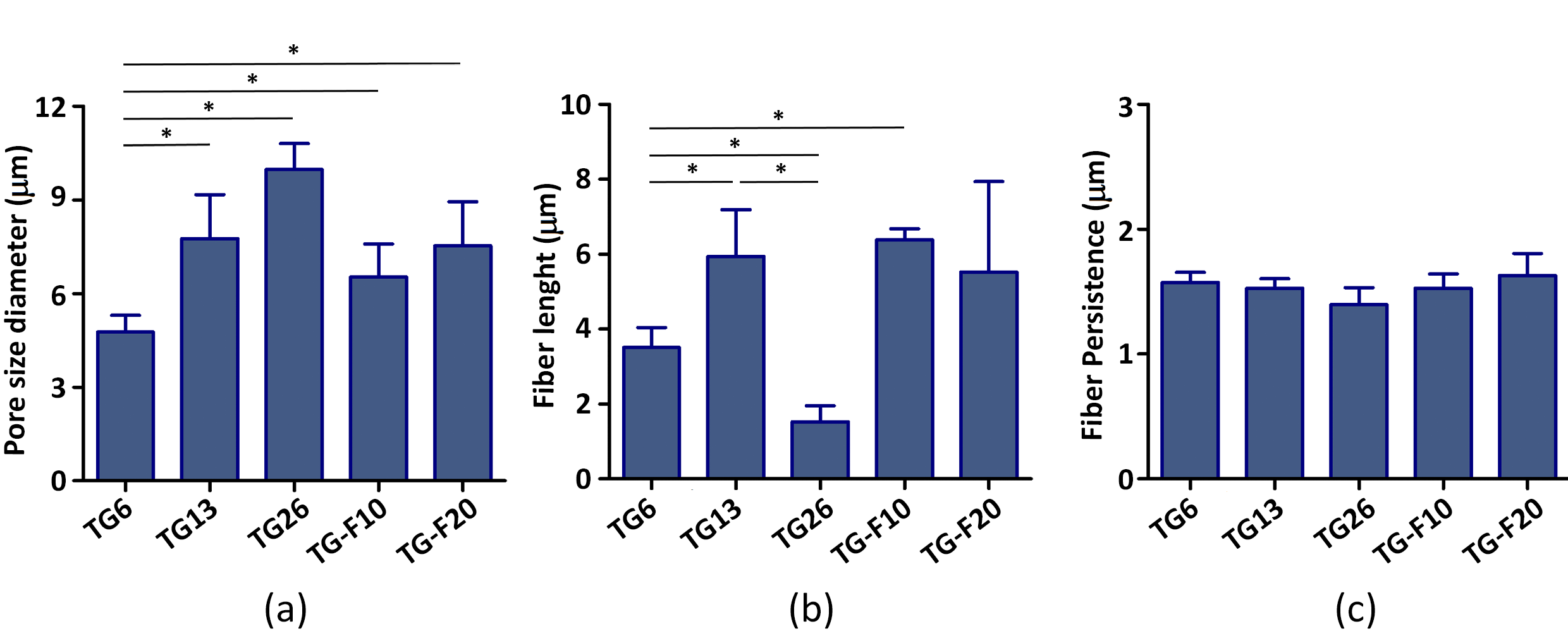

Supplement: S6 Fig — Average and standard deviation (std) of the morphological measurement obtained from Confocal Reflection Microscopy images. The number of samples used to calculate the Fiber length, Fiber persistence, and Pore size is nine (n = 3) since we analyzed three sub-images from each type. * Indicates statistically significant difference of non-parametric Mann-Whitney U-test (p<0.05). (TIF) [file pone.0171417.s006.tif]
