# Supplementary material for: Characterization of three-dimensional cancer cell migration in mixed collagen-Matrigel scaffolds using microfluidics and image analysis
Source: PLoS One. 2017 Feb 6;12(2):e0171417. doi: 10.1371/journal.pone.0171417 (PMC5293277; doi:10.1371/journal.pone.0171417)
Supplement: S1 Table — Average and standard deviation (std) of the morphological measurement obtained from the SEM images. The number of samples used to calculate the %Porosity, Fiber diameter and Number of pores and is three (n = 3) since we analyzed three images from each type. The number of samples used to calculate Pore size varied between sample types, since the unit used was the pore. Namely, the n values were n = 1830 (C), n = 1012 (CM) and n = 487 (CM+). (DOCX) [file pone.0171417.s007.docx]

| **Hydrogel** | % Porosity | Pore size  μm^2^ | Fiber diameter  μm | Number of pores |
| --- | --- | --- | --- | --- |
| **C** | 54.16 (4.46) | 0.64 (2.69) | 0.35 (0.001) | 610 (114.03) |
| **CM** | 45.08 (5.90) | 0.87 (2.60) | 0.59 (0.05) | 337.3 (69.0) |
| **CM+** | 47.60 (4.58) | 1.04 (6.39) | 0.72 (0.10) | 162.3 (30.5) |
