# Supplementary material for: Characterization of three-dimensional cancer cell migration in mixed collagen-Matrigel scaffolds using microfluidics and image analysis
Source: PLoS One. 2017 Feb 6;12(2):e0171417. doi: 10.1371/journal.pone.0171417 (PMC5293277; doi:10.1371/journal.pone.0171417)
Supplement: S2 Table — Average and standard deviation (std) of the morphological measurement obtained from the Confocal Reflection Microscopy images. The number of samples used to calculate the Fiber length, Fiber persistence, and Pore size is nine (n = 9) since we analyzed nine sub-images from each type. (DOCX) [file pone.0171417.s008.docx]

| **Hydrogel** | Fiber persistence μm | Fiber length  μm | Pore size  μm |
| --- | --- | --- | --- |
| **C** | 1.65 (0.07) | 2.69 (0.05) | 4.39 (0.15) |
| **CM** | 1.56 (0.11) | 2.73 (0.24) | 6.10 (0.74) |
| **CM+** | 1.47 (0.06) | 2.87 (0.15) | 10.87 (1.60) |
