# Supplementary material for: Characterization of three-dimensional cancer cell migration in mixed collagen-Matrigel scaffolds using microfluidics and image analysis
Source: PLoS One. 2017 Feb 6;12(2):e0171417. doi: 10.1371/journal.pone.0171417 (PMC5293277; doi:10.1371/journal.pone.0171417)
Supplement: S3 Table — Mean and standard error (parenthesis) of accumulated distance (in microns) after 12 hours of migration, and speed of migration (in microns per hour) in hydrogels C, CM and CM+, with no chemo-attracting substance (Control) using serum containing medium, (20% FBS) or after conjugation with integrin-blocking antibodies (20% FBS + Anti-β1, 20% FBS + Anti-β3 and 20% FBS + Anti-β1+β3). (DOCX) [file pone.0171417.s009.docx]

| **Hydrogel** | C | CM | CM+ |
| --- | --- | --- | --- |
| **Control**  MAD  Speed | 35.85 (2.44)  2.91 | 58.31 (2.34)  4.86 | 31.69 (1.95)  2.64 |
| **20% FBS**  MAD  Speed | 52.51 (2.17)  4.37 | 63.81 (1.87)  5.32 | 36.98 (1.82)  3.08 |
| **20% FBS + Anti-β1**  MAD  Speed | 45.93 (1.95)  3.83 | 46.48 (1.85)  3.87 | 42.94 (2.69)  3.58 |
| **20% FBS + Anti-β3**  MAD  Speed | 43.39 (2.48)  3.61 | 41.53 (1.80)  3.46 | 44.15 (2.69)  3.68 |
| **20% FBS + Anti-β1+β3**  MAD  Speed | 49.93 (2.90)  4.16 | 56.62 (1.90)  4.72 | 47.79 (2.44)  3.98 |
