# Supplementary material for: Characterization of three-dimensional cancer cell migration in mixed collagen-Matrigel scaffolds using microfluidics and image analysis
Source: PLoS One. 2017 Feb 6;12(2):e0171417. doi: 10.1371/journal.pone.0171417 (PMC5293277; doi:10.1371/journal.pone.0171417)
Supplement: S4 Table — Mean and standard error (parenthesis) of number of invading cells in C, CM, and CM+, hydrogels, and in hydrogels with Matrigel only at equal concentration as in CM (M, 2mg/ml) and CM+ (M+, 4mg/ml). The number of replicas of each experiment is 8 for 20%FBS, and 4 for serum free, Control experiments. (DOCX) [file pone.0171417.s010.docx]

| **Hydrogel** | C | M | CM | M+ | CM+ |
| --- | --- | --- | --- | --- | --- |
| **Control** | 0 (0) | 2.62 (0.82) | 0 (0) | 0 (0) | 0 (0) |
| **20% FBS** | 26.25 (5.28) | 1227.5 (49.85) | 560.62 (48.94) | 399.37 (40.21) | 186.42 (115.68) |
