# Supplementary material for: Characterization of three-dimensional cancer cell migration in mixed collagen-Matrigel scaffolds using microfluidics and image analysis
Source: PLoS One. 2017 Feb 6;12(2):e0171417. doi: 10.1371/journal.pone.0171417 (PMC5293277; doi:10.1371/journal.pone.0171417)
Supplement: S5 Table — Average (avg) and standard deviation (std) of the storage (G’) and loss (G”) moduli, both in pascal units (Pa) of the control, Matrigel only containing hydrogels. The standard deviation corresponds to three repetitions of each experiment (n = 3). (DOCX) [file pone.0171417.s011.docx]

| **Hydrogel** | G’ avg (std) | G’’ avg (std) |
| --- | --- | --- |
| **M** | 2.07 (1.10) | 0.28 (0.1) |
| **M+** | 10.99 (1.81) | 1.93 (1.92) |
