# Supplementary material for: Characterization of three-dimensional cancer cell migration in mixed collagen-Matrigel scaffolds using microfluidics and image analysis
Source: PLoS One. 2017 Feb 6;12(2):e0171417. doi: 10.1371/journal.pone.0171417 (PMC5293277; doi:10.1371/journal.pone.0171417)
Supplement: S6 Table — Mean and standard error (parenthesis) of accumulated distance (in microns) after 12 hours of migration in collagen only hydrogels with increasing crosslinking levels TG6, TG13, and TG26, and with increasing levels of fibronectin TG-F5, TG-F10 and TG-F20, with no chemo-attracting substance (Control) and using serum containing medium, (20% FBS). The numbers are average values obtain in two migration experiments. (DOCX) [file pone.0171417.s012.docx]

| **Hydrogel** | TG6 | TG13 | TG26 | TG-F10 | TG-F20 |
| --- | --- | --- | --- | --- | --- |
| **Control**  MAD  (sem) | 44.16  (2.08) | 47.56  (2.72) | 50.27  (2.61) | 48.44  (2.38) | 36.46  (2.21) |
| **20% FBS**  MAD  (sem) | 66.64  (5.57) | 82,20  (4.66) | 86.29  (5.25) | 55.56 (3.07) | 56.15  (3.79) |
